# Lack of correlation between the levels of soluble cytotoxic T-lymphocyte associated antigen-4 (CTLA-4) and the CT-60 genotypes

**DOI:** 10.1186/1740-2557-2-8

**Published:** 2005-10-31

**Authors:** Sharad Purohit, Robert Podolsky, Christin Collins, Weipeng Zheng, Desmond Schatz, Andy Muir, Diane Hopkins, Yi-Hua Huang, Jin-Xiong She

**Affiliations:** 1 Center for Biotechnology and Genomic Medicine, Medical College of Georgia, CA4095 Augusta, GA 30912; 2 Department of Pediatrics, University of Florida, Gainesville FL 32607, USA

## Abstract

**Background:**

Cytotoxic T lymphocyte-associated antigen-4 (CTLA-4) plays a critical role in downregulation of antigen-activated immune response and polymorphisms at the *CTLA-4 *gene have been shown to be associated with several autoimmune diseases including type-1 diabetes (T1D). The etiological mutation was mapped to the CT60-A/G single nucleotide polymorphism (SNP) that is believed to control the processing and production of soluble CTLA-4 (sCTLA-4).

**Methods:**

We therefore determined sCTLA-4 protein levels in the sera from 82 T1D patients and 19 autoantibody positive (AbP) subjects and 117 autoantibody negative (AbN) controls using ELISA. The CT-60 SNP was genotyped for these samples by using PCR and restriction enzyme digestion of a 268 bp DNA segment containing the SNP. Genotyping of CT-60 SNP was confirmed by dye terminating sequencing reaction.

**Results:**

Higher levels of sCTLA-4 were observed in T1D (2.24 ng/ml) and AbP (mean = 2.17 ng/ml) subjects compared to AbN controls (mean = 1.69 ng/ml) with the differences between these subjects becoming significant with age (p = 0.02). However, we found no correlation between sCTLA-4 levels and the CTLA-4 CT-60 SNP genotypes.

**Conclusion:**

Consistent with the higher serum sCTLA-4 levels observed in other autoimmune diseases, our results suggest that sCTLA-4 may be a risk factor for T1D. However, our results do not support the conclusion that the CT-60 SNP controls the expression of sCTLA-4.

## Background

Effective T cell activation requires a 'costimulation' signal that is mediated through CD28 interacting with B7 family members on antigen presenting cells (APC) [1]. The cytotoxic T lymphocyte associated antigen-4 (CTLA-4) was initially described as a B7 binding protein and a receptor expressed on the surface of activated T cells [2]. It belongs to the immunoglobulin gene superfamily and shares homology with CD28. CTLA-4 has been reported to be an important negative regulator of autoimmune diseases [3,4]. CTLA-4 blockade enhances T cell responses *in vitro *and *in vivo *[5,6], augments antitumor immunity [7] and exacerbates autoimmune diseases [8]. Several reports have indicated that CTLA-4 deficient mice show a severe lymphoproliferative disorder and autoimmune disease with early lethality [9,10]. Treatment with anti-CTLA-4 mAb of BDC2.5/NOD mice provoked a rapid onset of diabetes, indicating that a higher CTLA-4 presence was required for suppression of autoimmune phenomenon in these mice [11,12]. Recently, a soluble form of CTLA-4 (sCTLA-4) was found to be expressed constitutively by unstimulated human T cells [13]. Circulating sCTLA-4 protein was found to be present in human serum and is shown to possess an inhibitory effect on mixed leucocyte response [14].

Several studies have demonstrated a genetic association between polymorphisms within or near the *CTLA-4 *gene and T1D [15-19] as well as other autoimmune diseases [20-24]. This susceptibility locus has been recognized as *IDDM12*. Our previous studies indicated that *CTLA-4 *was the only gene contained in the *IDDM12 *susceptibility interval, suggesting that *CTLA-4 *is indeed the *IDDM12 *gene [16]. In a recent report by Ueda et al [25] the susceptibility interval was further narrowed to a 6.1 kb region at the 3' UTR of the *CTLA-4 *gene and the CT60-A/G single nucleotide polymorphism (SNP) was suggested to be the etiological mutation. The susceptible CT60-G allele was reported to produce a lower amount of soluble CTLA-4 mRNA in the peripheral blood lymphocytes than the disease resistant CT60-A allele. These results suggested that sCTLA-4 may confer protective effect against T1D. If this effect is indeed true, one would predict lower sCTLA-4 in the serum in T1D patients compared to controls. However, the prediction is in direct conflict with the observations in other autoimmune diseases including autoimmune thyroid disease [26], systemic lupus erythematosus [27] and myasthenia gravis [28], in which the serum sCTLA-4 levels are increased in patients compared to controls. The measurement of serum sCTLA-4 protein in a larger sample set is vital in evaluating the potential role of sCTLA-4 in T1D, and to better understand the molecular and functional basis underlying the genetic association between the *CTLA-4 *gene and T1D.

## Methods

### Patient sera

The study population consists of 218 subjects from the South-eastern United States. All study subjects were genotyped for HLA-DQB1 and evaluated for three autoantibodies (IA-2A, GADA and IAA) using established methods [29,30] Subjects used in this study are participants in the prospective assessment in newborns for diabetes autoimmunity (PANDA) program. Briefly, PANDA screens newborns from the general population as well as children with a first degree relative with T1D using HLA genotyping. Those subjects with high risk genes are monitored for the appearance of islet autoantibodies and clinical diabetes. Therefore, most of the autoantibody-negative (AbN) subjects also have high risk HLA genes and the AbN group is not randomly selected from the general population. The autoantibody-positive (AbP) subjects have been tested persistently positive for two or more islet autoantibodies. Based on our results from PANDA and previous studies, the AbP group has 70–80% of chance to progress to T1D [31] and indeed represent a very high risk group. Since autoantibody production is one of the hallmarks of autoimmunity, the AbP and T1D group can be combined to assess the impact of autoimmunity on the CTLA-4 levels. Appropriate institutional review boards approved the study design and informed consent was obtained from all subjects.

### Assay of sCTLA-4

A sandwich ELISA assay as described by Oaks et al [26] was used to measure the serum sCTLA-4 levels in a total of 218 subjects, including 117 autoantibody-negative (AbN), 19 autoantibody-positive (AbP) and 84 patients with T1D. The 96-well microtiter plates (Pierce Biotechnology, Rockford, IL) were coated with 1.0 ug/ml anti-CTLA-4 monoclonal antibody (clone BNI3; Pharmingen, San Deigo, CA). After blocking, 100 ul of 1:10 diluted serum samples were added to each well and the plates were incubated for 2 hr in a humid chamber at 37°C and then washed to remove unbound material. After washing, 100 ul biotinylated anti-CTLA-4 mAb (1.0 ug/ml, clone AS-33, Antibody Solutions, Palo Alto, CA) was added and the reactions were incubated for another 1 hr at 37°C in a humid chamber. Reactions were developed using streptavidin-peroxidase complex (Biorad, Hercules, CA) and 3,3',5,5'-tetramethy benzidine substrate (Sigma, St. Louis, MO) for 10 min at room temperature, the reaction was terminated with 2N H_2_SO_4 _and optical density was read at 450 nm and 630 nm. A standard curve was generated using a dilution series of commercially available CTLA-4-Ig fusion protein (0.125 ng/ml to 10 ng/ml Ancell, Bayport, MN). This assay has a linear range between 0.5 and 10 ng/ml and the vast majority of the samples fall under this range. Each sample was analyzed in duplicate.

### Genotyping of 3' untranslated region of CTLA-4 gene

A fragment of 268 bp encompassing the CTLA-4 C/T-60 single nucleotide polymorphism (SNP) in the 3' untranslated region was amplified using the forward primer 5'GCTTCATGAGTCAGCTTTGC3' and reverse primer 5'ATAGGACCACAGGT3'. The amplified PCR products were digested using the 10 units of HpyCH4IV (New England Biolabs) and separated on 3% agarose gels. The C-60 allele yielded two bands of 151 and 103 bp and the T-60 allele yields a band of 268 bp. The genotyping technique for the C/T-60 SNP was further confirmed by DNA sequencing of a subset of samples using a 300XL DNA sequencer (ABI Sciex).

### Statistical Analysis

Absorbance values obtained at 450 nm were normalized with the absorbance values at 630 nm. sCTLA-4 levels were log-transformed prior to analysis. Two of the T1D subjects had very high sCTLA4 levels with serum CTLA4 levels were > 12 ng/ml, or 3.5 standard deviations from the mean of 2.57 ng/ml. Further, initial analyses involving analysis of variance (ANOVA) indicated these two subjects' values were outliers. As such, the data for these two subjects were removed from all subsequent analyses. We used linear-mixed model ANOVA (Proc Mixed procedure of SAS) in which plate was included as a random effect to examine differences in sCTLA4 levels. Initially we analyzed phenotypic group (AbN, AbP, and T1D) alone, but subsequently conducted separate analyses adding other factors. These analyses were as follows: (1) factorial ANOVA with phenotypic group and CTLA-4 genotype; (2) factorial ANOVA with phenotypic group and gender; (3) ANOVA with phenotypic group and age as a covariate, and the interaction between age and phenotypic group; (4) ANOVA with phenotypic group and duration of T1D as a covariate, and the interaction between duration of T1D and phenotypic group; and (5) factorial ANOVA with phenotypic group and HLA genotype.

## Results

Clinical and demographic information is presented in Table 1. A majority of the subjects in the study were below the age of 20 years (73%) in all three groups. The subjects were tested for IAA, GADA and IA-2A autoantibodies as well as HLA-DQB1 genotypes. HLA-DQB1 genotyping information was available for 96.58, 94.74 and 98.78 percent of patients from the AbN, AbP and T1D groups, respectively. Eighty-eight percent of T1D subjects were diagnosed with T1D before the age of 20, with an average age of 8.8 (range 0.9–41.2). Fifty-seven out of eighty-two T1'1D subjects have a T1D duration of five years and less.

**Table 1:** Clinical and Demographic characteristics of subjects involved in the study with respect to number of individuals, age of diagnosis, duration of disease, genotype and antibody number.

Variable	AbN	AbP	T1D
Total number	117	19	82
Age (years)	15.1	14.5	15.8
**75%ile**	**22.0**	**28.5**	**20.1**
**90%ile**	**37.0**	**37.0**	**36.0**
Age range	(0.8–48.6)	(0.6–44.0)	(0.9–43.7)
			
Age of Diagnosis (years)			8.8 (0.8–41.2)
			
Duration of T1D (years)			6.2 (0–41.0)
			
**Age: n (percent)**			
<20 years	86 (73.51)	14 (73.68)	60 (73.17)
>20 years	31 (26.49)	5 (26.32)	22 (26.83)
			
**Sex: n (percent)**			
Male	53 (45.30)	9 (47.37)	50 (60.97)
Female	64 (54.70)	10 (52.63)	32 (39.03)
			
Genotype: n (percent)*			
0201/0201	8 (6.84)	0 (0.00)	13 (15.85)
0302/0302	12 (10.26)	4 (21.05)	6 (7.32)
0201/0302	31 (26.50)	5 (26.32)	38 (46.34)
0201/x	21 (17.95)	3 (15.79)	10 (12.20)
0302/x	23 (19.66)	4 (21.05)	11 (13.41)
x/x	18 (15.38)	2 (10.53)	3 (3.66)

Age, age of diagnosis and duration of disease is presented as means (range).

*Genotyping information was not available on one AbP, one T1D and 4 AbN individual(s).

Sex and genotype are presented as number of individuals of individuals (n). Values presented in parenthesis are percentage of total.

We first compared the serum sCTLA-4 levels between the three phenotypic groups (i.e., AbN, AbP and T1D). The protein levels for T1D (mean = 2.24 ng/ml, range = 0–10.1 ng/ml) and AbP (mean = 2.17 ng/ml, range = 0.2–7.7 ng/ml) were slightly higher than that in AbN (mean = 1.69 ng/ml, range = 0.0–11.5 ng/ml) (Table 2), although these differences were not statistically significant.

**Table 2:** sCTLA-4 levels in AbN, T1D and AbP individuals. Values presented are mean and 95% confidence interval in ng/ml.

Group	AbN	AbP	T1D	T1D/AbN	AbP/AbN
**All subjects (p = 0.58)**	1.7 (0.6–3.6) (n = 117)	2.2 (0.5–5.5) (n = 19)	2.2 (0.9–4.6) (n = 82)	1.3	1.3
CTLA-4 genotype					
A/A	1.9 (0.7–4.1) (n = 12)	2.5 (n = 1)	1.9 (0.8–4.9) (n = 19)	1.0	1.2
A/G	1.5 (0.5–4.0) (n = 21)	1.7 (0.4–4.2) (n = 7)	1.9 (0.7–3.8) (n = 16)	1.2	1.1
G/G	1.6 (0.7–3.0) (n = 49)	2.5 (0.9–5.4) (n = 9)	2.4 (1.1–4.4) (n = 26)	1.3	1.3
Male (p = 0.53)	1.7 (0.9–2.8) (n = 53)	2.3 (0.9–4.5) (n = 9)	2.3 (1.3–3.7) (n = 51)	1.4	1.3
Female (p = 0.70)	1.7 (0.9–2.8) (n = 64)	2.1 (0.8–4.2) (n = 10)	2.2 (1.2–3.6) (n = 31)	1.3	1.2
DQB1*0201 positive	1.3 (0.6–2.4) (n = 60)	1.8 (0.6–4.0) (n = 8)	2.2 (1.1–3.8) (n = 62)	1.7	1.4
DQB1*0201 negative	2.2 (1.2–3.8) (n = 53)	3.3 (1.4–6.8) (n = 10)	1.9 (0.8–3.8) (n = 19)	0.9	1.5
DQB1*0302 positive	1.6 (0.8–2.9) (n = 66)	2.4 (1.0–4.6) (n = 13)	2.3 (1.2–3.9) (n = 56)	1.4	1.5
DQB1*0302 negative	1.9 (0.9–3.3) (n = 47)	2.6 (0.9–5.9) (n = 5)	1.8 (0.7–3.6) (n = 25)	1.0	1.4

The serum CTLA-4 levels were analyzed after stratification by phenotypic groups (T1D, AbP and AbN) and the CTLA-4 CT-60 SNP genotypes (A/A, A/G and G/G) (Table 2). A mixed model ANOVA using phenotypic group and CT-60 genotypes as factorial fixed effects revealed no differences in sCTLA-4 levels between CTLA-4 genotypes (p = 0.46) or genotype/phenotype interactions (p = 0.82). A similar ANOVA using CT-60 genotypes alone as a fixed effect did not reveal any significant differences in sCTLA-4 levels between CTLA-4 genotypes (p = 0.64).

**Figure 1:**
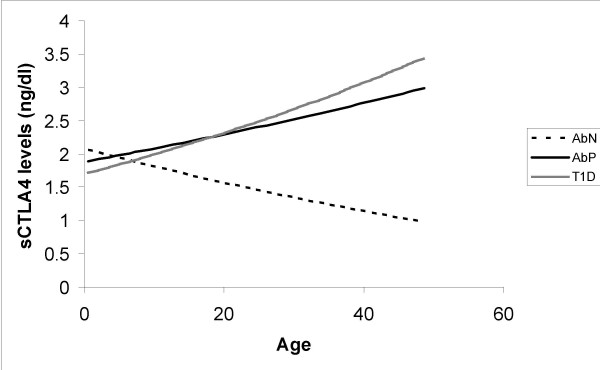
Relationship of sCTLA-4 with age and phenotypic groups. The lines shown are the estimated line based on the mixed linear model.

We then analyzed the data after conditioning on genetic, phenotypic or demographic parameters. Neither gender showed differences in serum CTLA-4 levels between the three phenotypic groups (Table 2). The relationship between sCTLA-4 levels and age differed between the three phenotypic groups (p = 0.022). The sCTLA-4 levels decreased with age in the controls (p = 0.048; Fig. 1). In contrast, sCTLA-4 levels increased with age in both the T1D and AbP groups (Fig. 1), although these relationships were not significant (p > 0.1). This difference in the relationship with age will result in AbN controls having lower sCTLA-4 levels at later ages compared with both AbP and T1D subjects. Serum sCTLA-4 levels in T1D subjects did not show an association with duration of disease (p = 0.4) nor with the age at disease onset (p = 0.6; data not shown).

The serum CTLA-4 levels were also analyzed after conditioning on the HLA-DQB1 genotypes by using phenotypic group, HLA-DQB1*201, and HLA-DQB1*302 as factorial fixed effects in a mixed model ANOVA. No differences were observed in sCTLA-4 levels between HLA-DQB1*0302 genotypes (p = 0.96), and the three phenotypic groups stratified by HLA-DQB1 genotype did not show any differences (Table 2, p = 0.51). AbN subjects with the DQB1*201 allele tended to have lower sCTLA levels (1.3 ng/ml vs 2.2 ng/ml), although the difference was not significant, a similar trend was observed in AbP group (1.8 ng/ml vs 3.3 ng/ml). The T1D group subjects with and without DQB1*201 allele have a very similar levels of sCTLA-4 (2.2 ng/ml vs 1.9 ng/ml). The differences between the three phenotypic groups for subjects with the DQB1*201 allele were not significantly different from the differences observed for those without the DQB1*201 allele (Table 2; p = 0.13). The main effect of the DQB1*0201 allele in the factorial mixed-model ANOVA was marginally significant (p = 0.08) with serum CTLA-4 levels being lower in individuals with a DQB1*0201 allele (mean = 1.8 ng/ml) than individuals without a DQB1*0201 allele (mean = 2.4 ng/ml). We decided to redo this analysis by combining the AbP and T1D groups for three reasons: (1) sample sizes were small for some of the genotype/phenotype combinations; (2) subjects that are positive for multiple antibodies and with a high risk HLA genotype are much more likely to develop T1D in future; and (3) autoimmunity is the common denominator of the AbP and T1D groups. When the data were analyzed with these two phenotypic groups considered as a single group (AbP + T1D vs. AbN), the main effect of DQB1*0201 allele became significant (p = 0.02), with the remaining effects still not significant. T1D and AbP subjects did show a trend towards having larger sCTLA-4 levels (mean = 2.2 ng/ml) compared to the AbN subjects (mean = 1.3 ng/ml) when only subjects with the DQB1*0201 were considered, however, the difference was not statistically significant (p = 0.15).

## Discussion

Type-1 diabetes is marked by the production of pancreatic islet β cell-specific auotantibodies and destruction of the insulin-producing β cells by autoreactive T cells. A role of CTLA-4 in the pathogenesis in T1D and other autoimmune diseases has been well documented. In this study we provide some suggestive evidence that high risk autoantibody positive subjects and T1D patients both have increased levels of sCTLA-4 in serum compared to autoantibody-negative subjects. We observed that larger differences in sCTLA4 levels between T1D/AbP and AbN subjects occur in the older age group. Further, we observed a difference between AbP/T1D subjects and AbN subjects for those carrying the DQB1*0201 allele.

As a negative regulator of T cell activation, blockade of CTLA-4 by monoclonal anti-CTLA-4 antibody provokes a rapid onset of diabetes in BDC2.5/NOD mouse model [11]. Treatment of animals with recombinant CTLA-4Ig molecule delays the onset of T1D and other autoimmune diseases [11,12,32-36]. However, the function and potential role of sCTLA-4 have not been well studied. sCTLA-4 is generated by alternative splicing of CTLA-4 mRNA, which induces a frame shift by deletion of a transmembrane region of CTLA-4 resulting in a native soluble protein [13]. sCTLA-4 is constitutively expressed on nonstimulated T cells and its expression is downregulated after T cell activation [14]. The soluble form of surface proteins is believed, in most cases, to play an inhibitory role due to competition for ligands with their surface counterparts. The finding that the sCTLA-4 expression level remains at sustained levels suggests that sCTLA-4 blocks the B7-mCTLA-4 interaction, thereby enhancing T-cell activation and autoreactivity by inhibiting the induction of anergy [37,38]. Alternatively, sustained sCTLA-4 levels may play a protective role via inhibition of the B7-CD28 interactions. Therefore, the role of sCTLA-4 in autoimmunity may depend on the relative binding affinity of sCTLA-4 to B7.1 and B7.2. This question was indirectly addressed in several autoimmune diseases by comparing the sCTLA-4 levels in the serum of patients and controls. Elevated sCTLA-4 has been reported in organ specific autoimmune thyroid disease [26], systemic lupus erythematosus [27] and myasthenia gravis [28]. These observations suggest that sCTLA-4 may contribute to the development of autoimmune diseases, probably through inhibiting the B7-mCTLA-4 interaction and down-regulation of T cell activation.

We are unaware of any study that has examined sCTLA-4 levels in the serum of T1D patients. A recent study by Ueda et al. [25], suggested that a SNP (CT60-A/G) in the 3' UTR of the CTLA-4 gene may determine the efficiency of the splicing and production of sCTLA-4 mRNA. Based on a small number of subjects, the susceptible G allele was suggested to produce lower amounts of sCTLA-4 mRNA. Based on these observations, the authors concluded that sCTLA-4 expression is the functional basis for the observed genetic association between T1D and the CTLA-4 gene. If this conclusion were correct, T1D patients would be expected to have lower serum CTLA-4 levels. Our results found no evidence to indicate that sCTLA-4 levels are decreased in patients compared to controls. In contrast, our data suggested that the serum sCTLA-4 levels were slightly higher in T1D patients. Our results also indicated that the increased sCTLA-4 levels in T1D patients were not due to the hyperglycemic conditions because the autoantibody positive subjects also had increased serum sCTLA-4 levels. We also directly tested the correlation between sCTLA-4 levels and the CT-60 SNP in all three phenotypic groups (AbN, AbP and T1D) and found no significant correlation in any of these groups. The discrepancies between our study and the previous report [25] may be explained by a number of factors. First, mRNA was studied in the previous report, while serum protein was analyzed in this study. As the biological function of sCTLA-4 is carried out at the protein level, our data is more applicable to the role of sCTLA-4 in T1D pathogenesis. Second, the sample size in the previous report [25] was extremely small and random variation is quite likely. Although the sample size in our study is not very large, it is several times larger than the previous report and is sufficiently powered to detect large differences. As there is no indication of a correlation between sCTLA-4 levels and the CT-60 genotype, it is unlikely that the C/T-60 SNP plays a major role in controlling the expression of sCTLA-4.

## Conclusion

Consistent with the observations in other autoimmune diseases in humans as well as data in the NOD mice, our data suggest that sCTLA-4 is potentially a risk factor for the development of T1D. Our data also raises a serious doubt about the conclusion that the expression of sCTLA-4 is controlled by the CT60 SNP in the 3' end of the CTLA-4 gene. Therefore, the functional basis for the genetic association between CTLA-4 and autoimmune diseases as well as the etiological mutation in the CTLA-4 region should be re-considered.

## Competing interests

The author(s) declare that they have no competing interests.

## Authors' contributions

SP and JXS designed the studies, helped with the interpretation and the writing of the manuscript. SP, CC and WZ were primarily involved in carrying out the clinical assessments and the acquisition of data. RP performed the statistical analyses and was involved in preparing the manuscript. AM and DS were responsible for collecting clinical samples and patient evaluation. DH and YH were involved in sample and data collection.
